# Estimation of concrete materials uniaxial compressive strength using soft computing techniques

**DOI:** 10.1016/j.heliyon.2023.e22502

**Published:** 2023-11-19

**Authors:** Matiur Rahman Raju, Mahfuzur Rahman, Md Mehedi Hasan, Md Monirul Islam, Md Shahrior Alam

**Affiliations:** a Department of Civil Engineering, International University of Business Agriculture and Technology, Dhaka, 1230, Bangladesh; b Department of Civil Engineering, Kunsan National University, 558 Daehakro, Gunsan, Jeollabugdo, 54150, Republic of Korea; c Department of Computer Science and Engineering, International University of Business Agriculture and Technology, Dhaka, 1230, Bangladesh; d Local Government Engineering Department (LGED), LGED Bhaban, Dhaka, 1207, Bangladesh

**Keywords:** Concrete compressive strength, Deep learning, Comparative analysis, Model optimization, Mix design

## Abstract

This study addresses a critical gap in concrete strength prediction by conducting a comparative analysis of three deep learning algorithms: convolutional neural networks (CNNs), gated recurrent units (GRUs), and long short-term memory (LSTM) networks. Unlike previous studies that employed various machine learning algorithms on diverse concrete types, our study focuses on mixed-design concrete and fine-tuned deep learning algorithms. The objective is to identify the optimal deep learning (DL) algorithm for predicting concrete uniaxial compressive strength, a crucial parameter in construction and structural engineering. The dataset comprises experimental records for mixed-design concrete, and models were developed and optimized for predictive accuracy. The results show that the CNN model consistently outperformed GRU and LSTM. Hyperparameter tuning and regularization techniques further improved model performance. This research offers practical solutions for material property prediction in the construction industry, potentially reducing resource burdens and enhancing efficiency and construction quality.

## 1 Introduction

Concrete ranks as the second most widely used construction material worldwide, owing to its abundant raw materials, ease of processing and handling, and ability to compact and withstand loads [1]. Over 30 billion tons of concrete are consumed annually, making it the most frequently used building material [2]. The world's largest concrete market, ready-mix concrete, is expected to generate about USD 600 billion in sales by 2025 [3]. It is a popular construction material made of cement, water, fine and coarse aggregates, and other additives. Its strength, durability, adaptability, and economy make it useful in civil, mechanical, and other tasks. The mechanical properties of concrete, especially compressive strength, must be investigated to ensure that engineered structures perform reliably and safely over their intended service life [4]. Moreover, precise ingredient proportioning is essential for cost-effective, high-quality concrete, and using proportioning design for predicting compressive strength is a highly effective approach.

A predictive model can be employed to estimate the compressive strengths of numerous concrete mix designs, facilitating the selection of those meeting the desired strength criteria for further physical testing [5]. This approach significantly reduces the number of trials required to achieve a specific compressive strength, resulting in substantial time and cost savings. Concrete compressive strength can be influenced by various factors, even in well-designed mixes, including raw material quality, variations in the water-cement ratio, mixing errors, inadequate curing, admixture-related issues, temperature fluctuations, mix design inaccuracies, quality control deficiencies, environmental conditions, testing variability, and strength changes over time [6,7]. Furthermore, the conventional method of measuring concrete compressive strength through destructive testing involves creating cubic or cylindrical specimens and subjecting them to compression testing using specialized equipment after the prescribed curing period [8]. In addition, the non-destructive evaluations for concrete frequently provide imprecise values for its strength due to several factors. Material changes, surface conditions, and procedure constraints may impair concrete strength tests [9]. The final strength of concrete depends heavily on the mix design. An optimized mix design model requires numerous laboratory experiments for optimal concrete compressive strength, which can be time-consuming, error-prone, and expensive [10]. Since then, researchers have presented empirical regression approaches to predict concrete compressive strength. Unfortunately, the firm nonlinear relationship between concrete components and compressive strength makes it difficult to obtain an adequate regression expression for concrete compressive strength [11].

Traditional machine learning techniques have long been a research focus for concrete compressive strength prediction [[12], [13], [14]]. These methods have traditionally been utilized to tackle the complexities associated with concrete strength forecasting [[15], [16], [17]]. However, with the advancement of artificial intelligence (AI), DL techniques have gained prominence in various industries due to their capacity to provide more accurate predictions compared to traditional approaches [18,19]. While conventional machine learning (ML) models have been frequently used in concrete strength prediction, they often exhibit limitations in terms of prediction accuracy and robustness [20]. In contrast, DL approaches have become increasingly popular due to their ability to handle complex nonlinear interactions and achieve higher prediction accuracy, making them a preferred choice for concrete strength prediction.

Additionally, optimized deep learning techniques have effectively addressed optimization problems across multiple technical dimensions. Convolutional neural networks (CNNs) algorithm can analyze concrete samples because they process pictures well [21]. Recurrent neural networks (RNNs) like long short-term memory (LSTM) networks and gated recurrent units (GRUs) can model curing processes and time-related aspects because they handle sequential data well [22]. To estimate concrete's maximum compression strength with finite accuracy and weak robustness, ML methods are preferred. However, standard ML and shallow learning models forecast less accurately [23]. Nowadays, DL approaches are used extensively due to higher prediction accuracy than regular machine learning models, and it can handle complex nonlinear interactions [24]. Also, the optimization problems in multiple technical dimensions can be solved using optimized deep-learning approaches [25].

Using deep learning techniques for predicting concrete compressive strength has received limited attention in prior research endeavors. Notably, Chen et al. [26] connected the power of the CNNs model, specifically the Visual Geometry Group (VGG) architecture, and achieved an impressive accuracy rate of 98 % in concrete compressive strength prediction. This performance surpassed traditional machine learning models, with a notable 2 % and 12 % superiority over the random forest (RF) and support vector regression (SVR) models, respectively. Gogineni et al. [27] delved into predicting concrete compressive strength incorporating mineral admixtures, employing the LSTM algorithm. Their findings showcased the remarkable predictive capabilities of LSTM, boasting R^2^ values of 0.997 and 0.994 for training and testing, respectively, alongside an exceptionally low root mean squared error of 1.917. Consequently, LSTM emerged as an adept and effective technique for forecasting concrete compressive strength, clearly outperforming classification and regression trees (CART). In the context of waste foundry sand concrete (WFSC), the deep neural networks (DNNs) model exhibited exceptional performance, evidenced by R^2^-values of 0.996 (training), 0.999 (testing), and 0.997 (validation) for compressive strength estimation. In contrast, the gene expression programming (GEP) model demonstrated subpar accuracy in estimating compressive strength relative to other developed models, as evidenced by R^2^-values of 0.851, 0.901, and 0.844 for the training, testing, and validation sets, respectively [28]. These notable advancements underscore the potential of DL in enhancing concrete compressive strength prediction, offering more accurate and reliable results than conventional methodologies. The available literature suggests a scarcity of studies employing DL techniques for predicting concrete compressive strength in the context of selected mix designs. This scarcity is due to the challenges in acquiring comprehensive experimental data to build accurate models. Therefore, this study compares experimental data with various DL algorithms to predict concrete compressive strength, considering several significant influencing factors. The study gathered data from 165 different mixed designs data after conducting experiments. Subsequently, this collected data was utilized to develop three deep learning algorithms: CNNs, GRUs, and LSTM, all aimed at predicting concrete compressive strength. This choice is supported by the established effectiveness of these algorithms in predicting concrete properties, as documented in the relevant literature.

This study stands out because it compares CNNs, GRUs, and LSTM models for predicting concrete strength. Unlike earlier research that used various ML algorithms on different concrete types, this study focuses on mixed-design concrete and fine-tunes deep learning algorithms. It helps fill a gap in the research by telling us which DL algorithm works best for this type of concrete. Experimental investigations within construction materials involve a series of labor-intensive and resource-consuming procedures that span multiple stages [29]. These encompass the meticulous selection of construction materials, the precise preparation of samples to replicate real-world concrete mixtures, the crucial curing process to foster proper material development, and the subsequent comprehensive testing phase to assess properties, including compressive strength. Each step demands expertise, time, and significant financial investment [30].

For the construction industry, embracing innovative solutions such as AI promises to significantly enhance efficiency and effectiveness in dealing with these formidable challenges [15]. AI can revolutionize many aspects of this laborious process. It can optimize material selection by swiftly analyzing vast datasets to identify the most suitable components for specific applications. AI can also predict material properties, streamlining sample preparation by reducing the need for extensive trial and error [31]. Moreover, AI has the potential to accelerate and optimize the curing process by monitoring environmental conditions in real time and adjusting as necessary.

Furthermore, it can assist in automating the testing phase, improving accuracy and saving both time and labor [32]. Therefore, the primary aim of this research is to delve into the practicality of DL (a subset of AI) in predicting material properties. This research ultimately seeks to optimize processes and alleviate the resource burdens associated with conventional experimental methods. The influence of individual input parameters on the predicted compressive strength of concrete is also determined.

## 2 Materials and methods

### 2.1 Experimental dataset and strategy

**Fig. 1:**
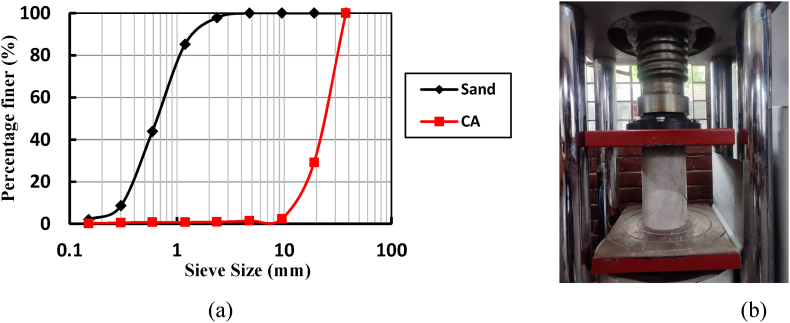
(**a**) Sieve analysis of sand and coarse aggregate (CA) and (**b**) UTM machine used for the compressive test.

This study meticulously assembled a substantial dataset comprising 165 experimental records on concrete compressive strength prediction. Among these records, 120 data points were sourced from the Local Government Engineering Division, Dhaka (LGED), Dhaka, while the remaining 45 underwent rigorous laboratory testing at IUBAT, Dhaka. This dataset revolved around concrete samples, encompassing five critical components within the 1 m³ concrete matrix: water, cement (specifically CEM II Ordinary Portland cement with a specific mass of 3.1 gr/cm³), fine aggregate, coarse aggregate, and an admixture. The mix design spanned a spectrum of water-cement (W/C) ratios, ranging from 0.38 to 0.68, with the water source adhering to ASTM D 1129 (1960) standards, drawn from Dhaka city's potable water supply. The aggregate selection included natural river sand and crushed gravel, with a maximum nominal size ranging from 12.5 mm to 20 mm. The specific gravity values for fine and coarse aggregates fell within 2.54–2.62 and 2.03–2.64, respectively. Precise particle size distribution curves were established for fine and coarse aggregates through manual crushing, aligning with ASTM C136/C136M − 14 [33] specifications, as depicted in Fig. 1. The maximum fineness modulus values for fine and coarse aggregates were recorded as 2.82 and 7.73, respectively, demonstrating consistent characteristics between natural and recycled coarse and fine aggregates.

Moreover, the concrete mix design underwent meticulous preparation, involving density and water absorption assessments following ASTM C 128–15 [34] and ASTM C 127–15 [35], respectively. This meticulous process also factored in necessary adjustments due to variations in specific gravity and water absorption characteristics. Subsequently, the freshly mixed concrete was meticulously cast into steel molds, left to set at ambient temperature for a 24-h, and then submerged in a water tank for comprehensive testing, strictly following ASTM C39 [36] standards. Mechanical strength tests were meticulously executed using a Universal Testing Machine (UTM, Fig. 1). The paper comprehensively presents detailed information regarding concrete mix proportions and offers a statistical summary of concrete parameter data essential for developing predictive models, all meticulously outlined in Table 1.

**Table 1:** Summary of statistical indication of the concrete parameters.

	Water (kg/m³)	Cement (kg/m³)	Fine Aggregate (kg/m³)	Coarse Aggregate (kg/m³)	Admixture (kg/m³)	Experimental Compressive Strength (MPa)
Count	165	165	165	165	165	165
mean	190.08	391.29	677.05	922.20	1.51	29.57
std	22.86	78.49	39.70	50.34	1.69	11.14
min	162.22	280.14	616.12	843.11	0.00	9.30
25 %	168.92	319.33	616.12	843.11	0.00	20.16
50 %	180.67	360.00	691.07	941.97	0.00	29.91
75 %	224.72	499.38	703.05	960.00	3.59	37.80
max	224.72	499.38	738.86	988.22	3.69	50.80

### 2.2 Data split and standardization

The database underwent a partitioning process to create two distinct subsets: a training set and a test set [16]. The purpose of this partitioning was to facilitate the development and evaluation of the predictive models reliably and effectively. The training set, which constituted 70 % of the experimental data (comprising 115 samples), served as the foundation for constructing and fine-tuning the predictive models [37]. Within this subset, the models learned to recognize patterns and relationships within the data, adjusting their internal parameters to optimize their predictive capabilities. In contrast, the test set was designed to be a separate, independent subset, encompassing 30 % of the data (consisting of 50 samples) [15]. This set was crucial for assessing the models' performance in a real-world scenario. By applying the trained models to this distinct dataset, researchers could effectively gauge how well the models could generalize their predictions to unseen data, which is a crucial measure of their reliability and effectiveness [38]. The utilization of both the training and test sets ensured that the models were not merely memorizing the data but genuinely learning and could provide accurate predictions for concrete compressive strength beyond the samples used during training. This partitioning approach is fundamental in developing robust and dependable predictive models. The training datasets played a pivotal role in developing algorithms tailored to the specific task of concrete strength prediction. These prediction algorithms provide insights into concrete strength characteristics, enabling informed decisions regarding concrete mix design and optimization. DL estimators can also handle features that mimic standard normal distributions with the statistical standardization measure. This method standardizes variables and yields dependable findings. The standard sample data score formula is Equation (1) [39].

(1) $$z-score=\frac{a-\mu }{s}$$

In Equation (1), *z* represents the standard score, *a* denotes the sample data, ***μ*** stands for the mean of the sample data, and *s* represents the standard deviation of the sample data. The resulting z-score indicates the degree to which the raw score deviates from the mean in units of standard deviations. A positive z-score indicates that the raw score is higher than the mean, while a negative z-score indicates that the raw score is lower than the mean. The reason for choosing the z-score technique is its ability to standardize data by scaling it to have a mean of 0 and a standard deviation of 1 [40]. This standardized data is beneficial when working with deep learning models as it helps them converge faster and can improve their performance. Additionally, the z-score technique is well-suited for data that follows a normal distribution since it centers the data around the mean and scales it according to the standard deviation [41]. This consideration was particularly relevant in this study, as the data pertains to concrete compressive strength, and such data often approximates a normal distribution.

### 2.3 Data distribution analysis

**Fig. 2:**
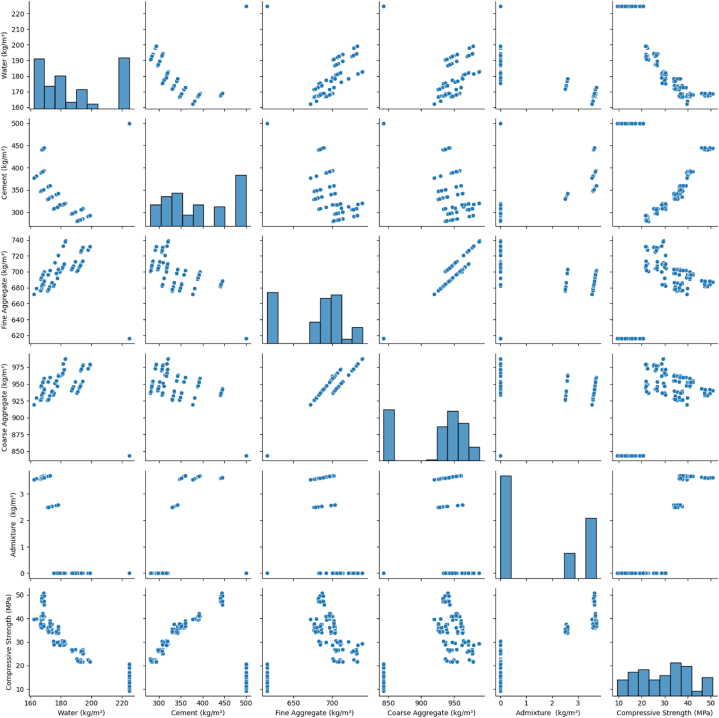
A pair scatter plot of the input parameters and strength.

In this study, the pair plot (Fig. 2) is a potent visualization method extensively utilized for data analysis, enabling a thorough exploration of the relationships among multiple input variables and experimental compressive strength. Moreover, the frequency distributions of individual input parameters are notably valuable in correlation analysis, as they furnish essential data that is ideally suited for robust DL modeling.

The noticeable gap in the histogram of admixture values, depicted in Fig. 2, raises questions about the database's sufficiency and reliability. While it does not necessarily indicate data unreliability, it underscores the importance of addressing data reliability and representativeness in the study. Depending on construction project requirements, admixtures in concrete are used selectively, leading to concentrated values and gaps in the dataset. This diversity in real-world concrete mix designs is expected. Future research could enrich the dataset with a broader range of concrete mixes, particularly those involving admixtures, addressing this observed gap and contributing to construction materials and deep learning applications in civil engineering.

### 2.4 Correlation coefficient analysis

**Fig. 3:**
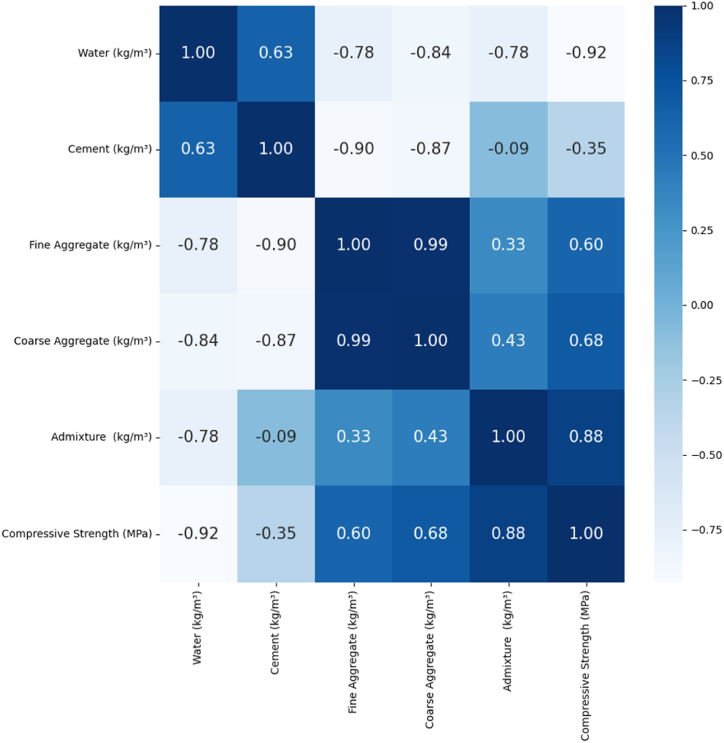
Pearson correlation analysis graph of input and output variables.

Fig. 3 illustrates the distribution of these variables, accompanied by Pearson correlation coefficients quantifying their relationships. The correlation values are color-coded in this representation, reflecting their numerical values within the [−1, 1] range [42]. A higher absolute coefficient denotes a stronger correlation, whereas values closer to 0 indicate a weaker association [43]. The Pearson correlation coefficients for this study between parameters revealed a mixed pattern, with some variables exhibiting strong positive associations, indicating significant positive relationships, while others displayed weak or negative correlations, suggesting limited or inverse connections among specific parameters. This comprehensive correlation analysis unveils the intricate interplay among variables, highlighting instances where factors positively reinforce each other. It also identifies variables with less overt influence or the potential for adverse effects within the dataset, providing valuable insights into the complex relationships at play. The correlation analysis demonstrates that experimental concrete compressive strength exhibits positive correlations with fine aggregate, coarse aggregate, and admixture. In contrast, it shows negative correlations with water and cement. Notably, the correlation between concrete compressive strength and admixture is strong, with a coefficient of 0.88, while the correlation between concrete compressive strength and water is the most negative, at −0.92. The Pearson correlation matrix analysis identifies correlations exceeding 0.80, indicating potential multicollinearity among variables [44]. This analysis, conducted using the standard scaler function in the Python sklearn library, provides valuable insights into the intricate relationships between input variables and the compressive strength of concrete in mix design.

### 2.5 Deep learning algorithms

**Fig. 4:**
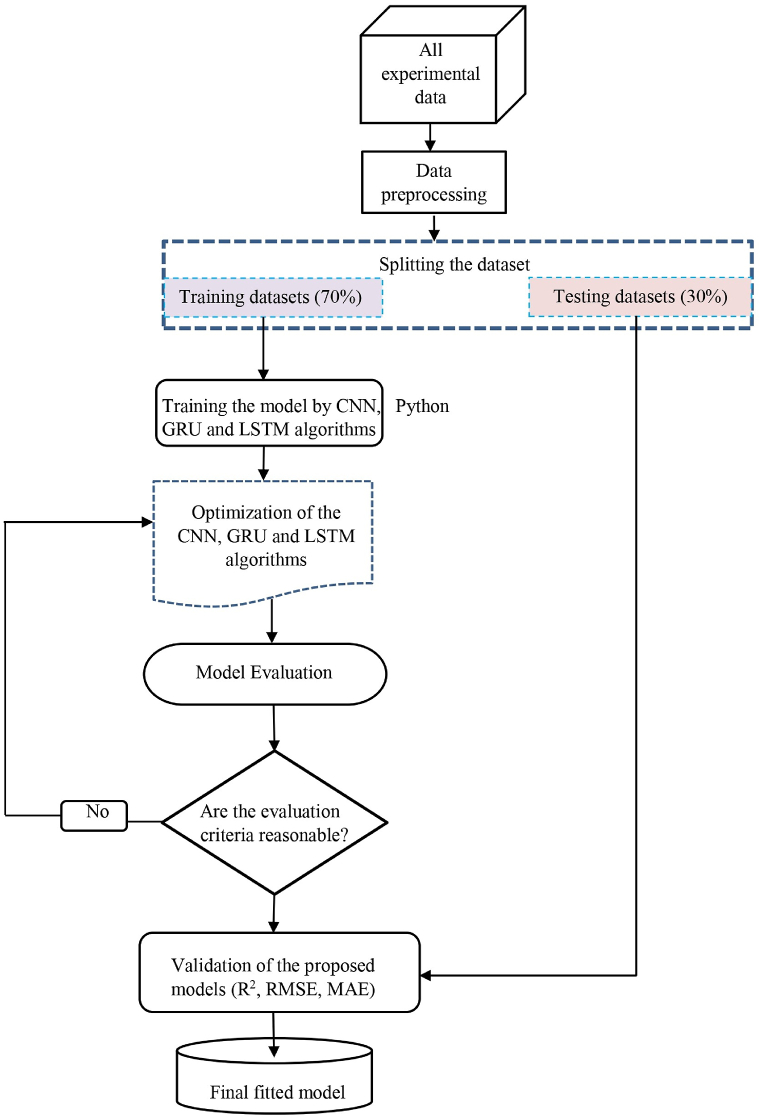
Schematic depiction of the proposed methodology.

Deep learning (DL) algorithms are increasingly used to predict concrete strength, offering improved accuracy in handling complex data [45]. These algorithms analyze factors like the concrete mix and how it's cured to predict its compressive strength. This application of DL not only enhances production processes but also improves quality control and safety in construction. In this study, three different models were used to predict concrete strength: the CNN model, the GRU model, and the LSTM model. The analysis was done using the Python programming language on the Google Colab platform. Fig. 4 provides a visual overview of the methodology used in this study, illustrating the step-by-step process and the connections between different components.

#### 2.5.1 Convolutional neural network

**Fig. 5:**
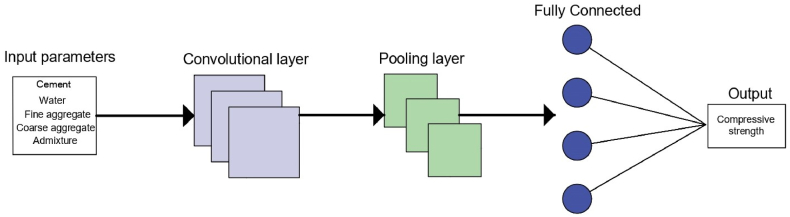
Illustration of a convolutional neural network (CNN) model.

The CNN algorithm has recently gained popularity for its utility in predicting concrete compressive strength (CS) [46]. CNN algorithm represents a modern deep learning framework comprising multiple layers adept at processing and reshaping input data to generate an output [47]. In the context of this study, our CNN architecture was constructed with a one-dimensional convolutional layer, a one-dimensional maximum pooling layer, a one-dimensional average pooling layer, and a fully-connected layer. Rectified Linear Unit (ReLU) was utilized for activation functions within each convolutional layer, while the optimization process was carried out using the Adam optimizer [48]. The CNN model consists of multiple layers, as depicted in Fig. 5. The convolutional layer primarily conducts data convolution by applying filters to the input data, leading to data transformation. The pooling layer performs essential subsampling, reducing computational complexity and mitigating potential overfitting issues [49]. Lastly, the fully connected layer plays a pivotal role in converting previously extracted features into the final output, serving as a critical component in the CNN architecture.

#### 2.5.2 Gated recurrent unit

Gated recurrent unit (GRU) algorithms, which belong to the category of recurrent neural networks (RNNs), are increasingly valuable in concrete strength prediction, primarily because of their adeptness in handling sequential data [50]. The workflow begins with data collection, involving the compilation of an extensive dataset that includes crucial elements like mix design variables and concrete strength measurements. After rigorous data preprocessing, the dataset is divided into training, validation, and testing subsets to enable comprehensive model assessment. Notably, GRU models are well-regarded for their efficient capacity to capture temporal dependencies in the data [51].

#### 2.5.3 Long short-term memory

The LSTM network, introduced by Hochreiter and Schmidhuber [52], was designed to address issues related to the “gradient disappearance” and “gradient explosion” in neural networks by introducing a gating mechanism. LSTM, known for its effectiveness in handling sequential data, can capture long- and short-term dependencies within time-series data, making it a valuable tool for feature extraction in time-series data, particularly in concrete strength prediction [53]. The process commences with data collection, involving the compilation of a dataset containing mixed design parameters, curing conditions, and concrete strength measurements. After thorough data preprocessing and the division of data into training, validation, and testing sets, the LSTM model's architecture is carefully configured to accommodate the sequential nature of the data. This precise utilization of LSTM models greatly enhances concrete production practices, aids in construction quality control, and ultimately contributes to the safety and durability of concrete structures.

#### 2.5.4 Evaluation criteria

Several metrics were used in some studies to evaluate the performed models and compare their robustness sufficiently. Accordingly, several statistical parameters such as R^2^, MSE, mean absolute percentage error (MAPE), RMSE, average bias error (MBE), t-statistic test (T stat), a20-index, and scatter index (SI) were used [54,55]. This study uses four frequently used performance indices, such as R^2^, RMSE, MAE, and a20-index for analyze the performance of selected DL models. R^2^ values closer to one indicate a better relationship between the desired result, but R^2^ values greater than 0.85 show a substantial relationship [56]. Both RMSE and MAE show the difference between expected and experimental values. RMSE is an efficient data change measure unaffected by predicted and actual value units and size. The MAE calculates average error by comparing experimental data to expected results. The “a20-index” is a straightforward yet meaningful statistical metric used to gauge the performance of predictive models, particularly in fields like engineering. It quantifies the percentage of samples for which a model's predictions fall within a ±20 % deviation from the actual experimental values [32,38]. Equations (2), (3), (4), (5) express these four assessment metrics mathematically [38,57].

(2) $$R^{2}=1\frac{\sum_{i=1}^{n}{\left(P_{y}-O\right)}^{2}}{\sum_{i=1}^{n}{\left(O-O\right)}^{2}}$$

(3) $$MAE=\frac{1}{n}\sum_{i=1}^{n}\left|\left(P_{y}-O\right)\right|$$

(4) $$RMSE=\sqrt{\frac{1}{n}\sum_{i=1}^{n}{\left[\left(P_{y}-O\right)\right]}^{2}}$$

(5) $$a20-index=\frac{m20}{M}$$

Where *n* is the number of observations, *P*_*y*_ is the value predicted by the model, *O* is the observed value, and $\bar{O}$ is the average of the experimental values. In addition, according to Equation (5), “M” represents the total number of samples in the dataset, and “m20” denotes the number of samples where the ratio of “observed value” to “predicted value” falls within the range of 0.80–1.20. It is worthy to note that in the case of a perfectly accurate predictive model, the “a20-index” should ideally equal 1. What makes the proposed “a20-index” valuable is its meaningful interpretation in the field of engineering, as it quantifies the percentage of samples for which the predicted values closely align with the experimental values, with a tolerance of ±20 % [15,32].

Moreover, the important analysis of input variables on the output is a critical step in predictive modeling, as it provides valuable insights into which factors exert the most significant influence on the target variable [58]. This understanding is instrumental in various applications, including optimizing processes, enhancing model interpretability, and guiding decision-making. In domains such as construction and concrete strength prediction, it aids engineers in identifying the key components of the mix that impact strength outcomes, enabling them to fine-tune mix designs for desired performance.

## 3 Results of the DL algorithms

### 3.1 Initial modeling

The CNNs, GRUs, and LSTM algorithms were developed using their default hyper-parameter values at first, and their performance was evaluated using prediction accuracy and error measures. Table 2 summarizes the statistical analysis of the R^2^, RMSE, MAE, SD, and a20-index results for predicting concrete strength using several DL algorithms on the training and testing datasets.

**Table 2:** Summary of statistical analysis results of the algorithms.

Data Set Type	DL Algorithms	R^2^	RMSE	MAE	SD	a20-index
Training Set	CNN	0.909	3.425	2.906	10.73	0.84
	GRU	0.899	3.625	3.051	10.66	0.88
	LSTM	0.889	3.621	3.009	10.77	0.73
Testing Set	CNN	0.907	3.147	2.609	10.54	0.86
	GRU	0.903	3.216	2.569	10.43	0.84
	LSTM	0.901	3.257	2.613	10.52	0.78

The CNN achieved the highest R^2^ (0.909) and the lowest RMSE (3.425), MAE (2.906), and SD (10.73) among the DL methods, suggesting the best overall performance on the training set. The GRU and LSTM models also performed admirably but with slightly larger errors and lower R^2^ values. Similarly, the CNN model had the highest R^2^ (0.907) and the lowest RMSE (3.147), MAE (2.609), and SD (10.54) on the testing dataset, showing higher prediction accuracy when compared to the other techniques. The GRU and LSTM models performed well but with significantly higher errors and lower R^2^ values.

In the context of statistical analysis for predicting concrete strength, the CNN model consistently exhibits superior performance when compared to the GRU and LSTM models. This observation holds for both the training and testing datasets. The CNN model demonstrates more excellent performance, as indicated by its higher R^2^ and lower RMSE, MAE, and SD values. Before optimization, the “a20-index” values range from 0.73 to 0.88, indicating that the model's predictive performance varied across different predictions, with some falling outside the ±20 % deviation from the actual values. These values suggest the model's accuracy in predicting concrete strength exhibited some inconsistency. The efficiency of the CNN can be due to its ability to extract features, recognize spatial patterns, and simulate the complex correlations between parameters of concrete mix and compressive strength [59]. Nevertheless, it is crucial to carefully analyze the dataset characteristics and problem details when choosing the most appropriate algorithm. This is because additional experimentation and optimization may be required to identify the ideal model for predicting concrete strength.

**Fig. 6:**
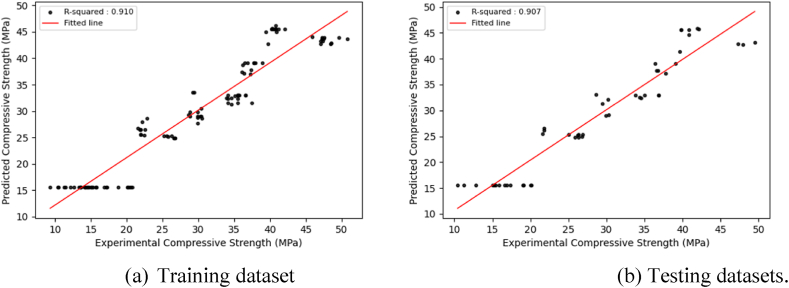
Regression plots between experimental and predicted compressive strengths using the CNN model.

**Fig. 7:**
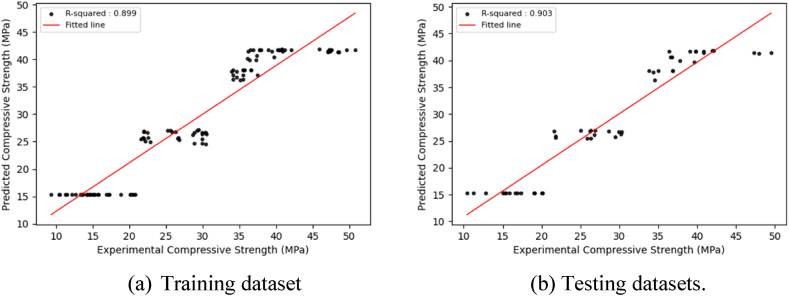
Regression plots between experimental and predicted compressive strengths using the GRU model.

**Fig. 8:**
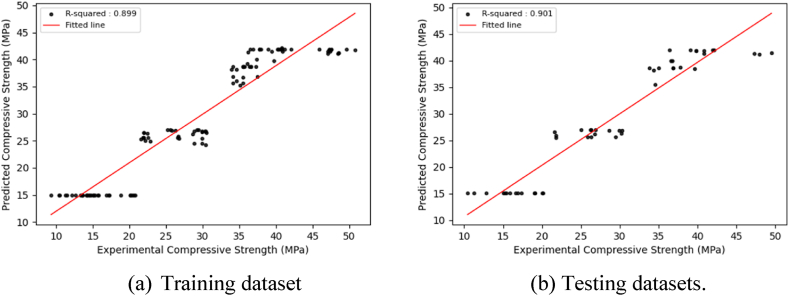
Regression plots between experimental and predicted compressive strengths using the LSTM model.

The main goal of model training is to develop a model that can successfully generalize and make accurate predictions on new, unknown data. This inquiry presents visualizations (Fig. 6, Fig. 7, Fig. 8, Table A1) that depict the distribution of projected findings compared to actual results for all models. These Figures effectively demonstrate the correlation between experimental concrete compressive strength and the predictions made by the three models. Throughout the training phase, it was seen that all three models encountered difficulties in effectively capturing the complex patterns present in the observation data. As a result, there was a significant increase in the correlation coefficient between the projected outcomes and the actual results. The CNN model exhibited robust correlation values during training and testing. The preliminary modeling indicates a satisfactory outcome in terms of prediction. However, utilizing the optimized algorithm, which will be thoroughly examined in the subsequent section, leads to further enhancements.

### 3.2 Model optimization with DL algorithms

Model optimization with deep learning (DL) algorithms is crucial for enhancing predictive performance and applicability [22]. DL models offer immense flexibility but require fine-tuning to achieve peak efficiency. Through hyperparameter tuning, regularization, and techniques like early stopping and ensemble learning, model optimization tailors DL models to the specific task [60]. Optimized DL models yield more accurate results in applications like concrete strength prediction, enabling precise control over mix designs and ensuring the safety and longevity of construction projects. This optimization involves striking the right balance between model complexity, generalization, and efficiency, ultimately delivering superior predictive accuracy and reliability [61].

In the process of optimizing the deep learning models for concrete strength prediction, mitigating overfitting concerns was a critical aspect. To address overfitting, various techniques were employed [32,37,62]. The primary approach involved regularizations, such as L1 and L2 regularization, which were applied during the model training phase. These regularization techniques help prevent the models from becoming too complex and fitting noise in the training data, thus improving their generalization to unseen data. Additionally, the use of techniques like early stopping was pivotal. By monitoring the model's performance on a validation dataset during training, early stopping allowed us to halt the training process when the model's performance on validation data started to degrade, preventing it from over-optimizing on the training dataset [63]. Ensemble learning was also employed, combining the strengths of multiple models to improve overall predictive accuracy while reducing the risk of overfitting. In the context of this study, no instances of overfitting were detected. This indicates that the deep learning models did not excessively fit the training data and maintained their ability to generalize to unseen data, ensuring the reliability of the predictions.

Table 3 presents selected properties of the CNN, GRU, and LSTM models before and after optimization. Initially, the CNN model used the Adam optimizer, 100 epochs, and a learning rate of 0.01. After optimization, it employed the Adam optimizer, extended to 200 epochs, with a reduced learning rate of 0.001. The GRU and LSTM models began with the SGD optimizer, 100 epochs, and a learning rate of 0.01, and following optimization, they switched to the Adam optimizer, ran for 200 epochs, and used a lower learning rate of 0.0001. These optimizations aimed to fine-tune the models for improved performance in concrete strength prediction.

**Table 3:** Summary of selected model properties before and after optimization.

			
CNN	Optimizer	Adam	Adam
	Epochs	100	200
	Learning Rates	0.01	0.001
GRU	Optimizer	SGD	Adam
	Epochs	100	200
	Learning Rates	0.01	0.0001
LSTM	Optimizer	SGD	Adam
	Epochs	100	200
	Learning Rates	0.01	0.0001

**Table 4:** Summary of statistical analysis results of the algorithms after optimization.

Data Set Type	DL Algorithms	R^2^	RMSE	MAE	SD	a20-index
Training Set	CNN	0.975	1.779	1.161	11.27	0.90
	GRU	0.976	1.758	1.090	11.27	0.89
	LSTM	0.973	1.753	1.079	11.27	0.89
Testing Set	CNN	0.968	1.831	1.312	10.48	0.90
	GRU	0.968	1.842	1.295	10.51	0.88
	LSTM	0.969	1.808	1.263	10.50	0.88

Based on the optimized DL models, the statistical analysis results of CNN, GRU, and LSTM demonstrate significant improvements in predictive accuracy based on the optimized DL models. All three models attain high R^2^ values in the training set, with CNN achieving 0.975, GRU 0.976, and LSTM 0.973, indicating strong predictive capabilities. In addition, their RMSE, MAE, and SD values are drastically reduced, which improves precision. In the testing set, the models maintain impressive R^2^ values from 0.968 to 0.969, demonstrating their ability to generalize well to unobserved data. In addition, the decreased RMSE, MAE, and SD values in the testing set indicate the models' ability to reduce prediction errors. After optimization, the “a20-index” values, ranging from 0.88 to 0.90, suggest that a substantial portion of the predictions falls within ±20 % deviation from the actual values, indicating relatively accurate predictive performance, with variations among individual predictions. These enhancements demonstrate the effectiveness of optimization in augmenting the models' accuracy and precision in predicting concrete compressive strength, which is essential in construction and structural engineering applications. The overview of prediction accuracy and evaluation metrics for the optimized deep learning models is presented in Table 4.

**Fig. 9:**
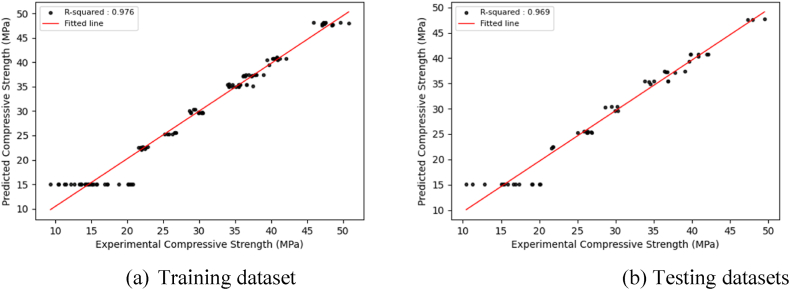
Regression plots between experimental and predicted compressive strengths using the optimized CNN model.

**Fig. 10:**
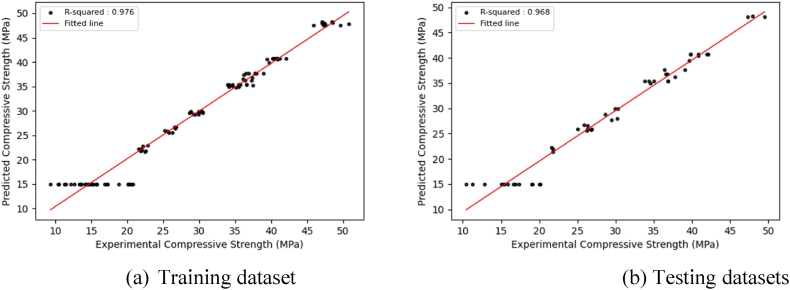
Regression plots between experimental and predicted compressive strengths using the optimized GRU model.

**Fig. 11:**
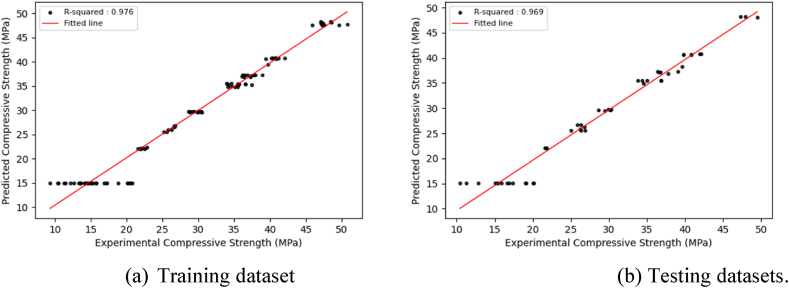
Regression plots between experimental and predicted compressive strengths using the optimized LSTM model.

The optimized DL models exhibit consistent prediction patterns in the training and test datasets, displaying minor deviations in prediction scores. The optimized DL models achieve high prediction accuracy and low error rates through hyperparameter adjustments for both the training and test datasets, resulting in improved model performance. Fig. 9, Fig. 10, Fig. 11, which correspond to CNNs, GRUs, and LSTM models, illustrate a close alignment between the best-fit line and the identity line, underscoring the precision of the model's predictions.

### 3.3 Comparison of results of DL algorithms

The testing dataset played a crucial role in this study as it was utilized for validation and conducting a comparative analysis of concrete compressive strength predictions. This dataset served as an independent set of concrete samples, separate from the training data used to develop the predictive models in this study. The model's accuracy and reliability were assessed by applying the trained DL algorithms to this testing dataset.

The statistical analysis of DL algorithms for concrete compressive strength prediction revealed substantial performance improvements after optimization. Initially, CNN, GRU, and LSTM models achieved R^2^ values of around 0.907, 0.903, and 0.901, respectively. Post-optimization, all three DL algorithms consistently achieved R^2^ values of approximately 0.968. Likewise, the RMSE values decreased significantly, with CNN, GRU, and LSTM models decreasing from approximately 3.147, 3.216, and 3.257 to 1.831, 1.842, and 1.808, respectively. Additionally, the MAE values showed a substantial decrease, with values of approximately 2.609, 2.569, and 2.613 for CNN, GRU, and LSTM models in the initial testing phase, reducing to 1.312, 1.295, and 1.263 after optimization. Among the DL algorithms exhibited low RMSE and MAE values, with CNN having the lowest RMSE of 1.831 and LSTM having the lowest MAE of 1.263. These results collectively suggest that all three DL models perform exceptionally well after optimization, making it challenging to declare one as the best. Instead, choosing the most suitable model may depend on specific project requirements or considerations, as all three have demonstrated impressive predictive capabilities.

**Fig. 12:**
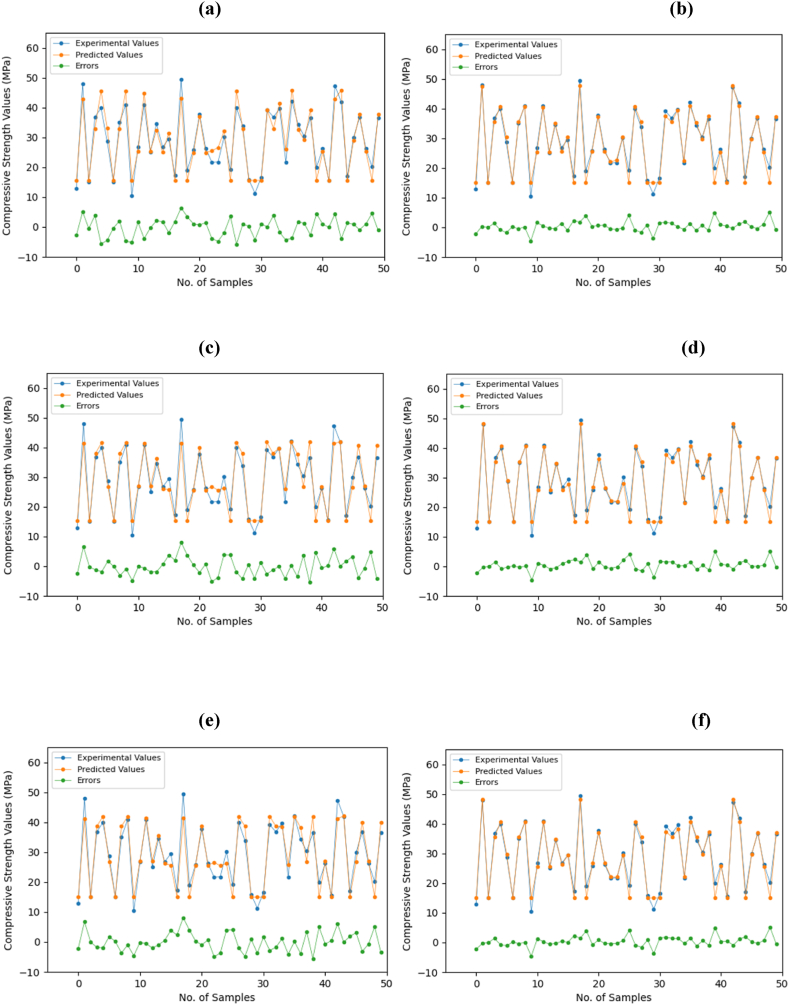
Comparison of measured compressive strength with relative errors for testing dataset; (a) CNN, (b) Optimized CNN, (c) GRU, (d) Optimized GRU, (e) LSTM, and (f) Optimized LSTM.

Fig. 12 shows the discrepancy between the observed data and the calculated results. Several different DL methods were utilized to identify the pattern concealed inside the experimental data. The predicted dataset's variance was compared to the DL models. The more significant the gap between the experimental dataset and the DL algorithms, the bigger the number of errors. In each graph, the experimental values are depicted by the lines that are dark blue, while the orange lines indicate the anticipated values. The green line underneath these lines shows the errors. In addition, the significant reduction in the error gap after optimization can be attributed to the fine-tuning and refinement of DL models. During the optimization process, various model hyperparameters, such as learning rates and epochs, were adjusted to enhance the model's ability to capture the underlying patterns in the experimental data.

### 3.4 Importance analysis of input variables on output

**Fig. 13:**
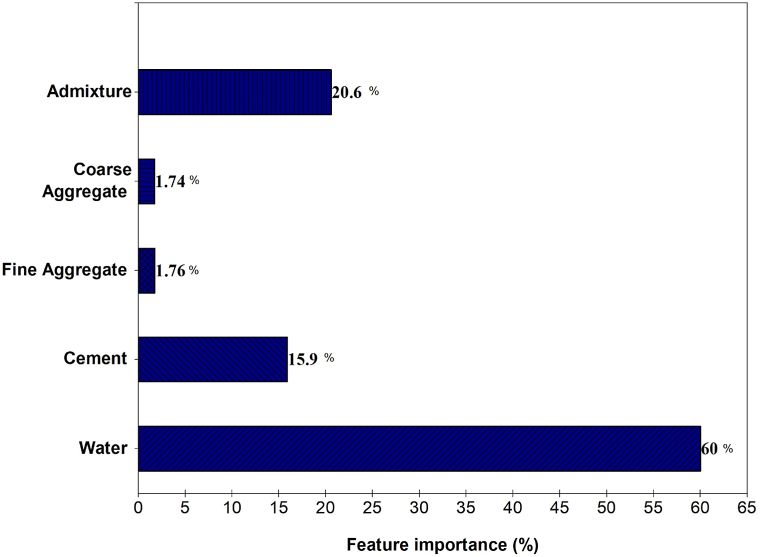
Input parameter's importance to concrete strength.

The importance analysis of input variables on the output, in this case, the composition of concrete mix components, plays a pivotal role in understanding their contribution to concrete strength [64]. Fig. 13 below displays all the features used in the compressive strength prediction model and their relative importance. Among the components, water holds the highest percentage at approximately 60 %, making it the most abundant in the mix. Following closely is the admixture, constituting about 20.6 % of the total mix. Cement is the next most substantial component at 15.91 %, while fine aggregate and coarse aggregate make up the remaining portions, each contributing approximately 1.74 % and 1.72 %, respectively. This study confirms that coarse aggregate has the least impact on predicting compressive strength, in line with previous research [65]. In the realm of concrete strength, it's crucial to understand that the water-cement ratio significantly affects compressive strength. More water generally means higher strength, but excessive water can lead to problems like increased porosity and reduced strength [66]. Thus, precise control of the water-cement ratio is essential for optimizing the mix. Additionally, the type and dosage of admixtures, including plasticizers or superplasticizers, introduce further dimensions for influencing both workability and strength [67]. Moreover, it should be recognized that the properties of aggregates, encompassing both fine and coarse varieties, contribute significantly to the overall mix volume and characteristics. By addressing these factors separately, concrete producers can customize their mix designs to meet specific strength and workability requirements while preventing potential issues associated with excessive water or other imbalances. This comprehensive approach to concrete mix design ensures the production of durable and high-quality concrete structures.

### 3.5 Limitations and future work

The study explores applying soft computing methods to predict uniaxial compressive strength in concrete materials. While the study offers valuable insights, it does present some limitations that should be considered. These limitations include the relatively small dataset, which could benefit from an increase in the quantity of data for improved robustness. Additionally, the absence of certain relevant variables and non-destructive test data may affect the comprehensiveness of the analysis. Furthermore, ensuring model generalization across diverse concrete compositions could be a point of improvement in this research. Despite these limitations, the study contributes to the field of concrete material analysis and provides a foundation for further research in applying soft computing techniques for strength estimation.

In terms of future studies, there is ample opportunity for further research in this domain. Future investigations could focus on expanding the dataset to encompass a more extensive range of concrete compositions, including variations in admixtures and mix designs. Including non-destructive test data and additional relevant variables would provide a more comprehensive foundation for predictive models. Moreover, research endeavors could explore advanced soft computing techniques and ensemble methods to enhance the accuracy and generalization of predictive models. This would contribute to a more robust and reliable framework for estimating uniaxial compressive strength in concrete materials, with potential applications in the construction and civil engineering industries.

## 4 Conclusions

This study developed a DL model for compressive strength prediction using a relatively small concrete mixture database. The optimized model displayed higher accuracy, reduced variability, and significant improvements in predictive performance. All DL models (CNN, GRU, and LSTM) initially demonstrated potential, with CNN leading the training set. Post-optimization, all three models achieved remarkable R^2^ values (∼0.968) and significantly reduced RMSE, MAE, and SD. Among the optimized models, GRU excelled as the best performer. Testing confirmed their ability to generalize. Optimization substantially decreased prediction errors, enhancing precision. The study underscored water, admixture, and cement's importance in concrete mix compositions and emphasized water's critical role, with the water-cement ratio being a vital determinant of desired properties. However, it is essential to acknowledge that model optimization in DL has its set of challenges. These include the intensive computational requirements, the potential risk of overfitting, the absence of a one-size-fits-all approach, reliance on data quality and quantity, increased model complexity, potential diminishing returns, the need for expert domain knowledge, and the possibility of converging to local optima. A careful and balanced approach is needed to address these challenges, considering both model performance and resource efficiency in line with specific task requirements. Moreover, the future scope of this study may involve further improving model efficiency by exploring alternative optimization techniques.

## Ethical approval

No ethical approval was required.

## Funding

No funding was received for conducting this study.

## Data availability statement

All data, models, or codes generated or used during the study are available from the corresponding author by request. Dataset link: https://github.com/Sirfowahid/Dataset2.

## CRediT authorship contribution statement

**Matiur Rahman Raju:** Writing – original draft, Formal analysis, Conceptualization. **Mahfuzur Rahman:** Writing – review & editing, Supervision, Formal analysis, Conceptualization. **Md Mehedi Hasan:** Writing – review & editing, Software. **Md Monirul Islam:** Writing – review & editing, Validation, Supervision. **Md Shahrior Alam:** Writing – review & editing, Data curation.

## Declaration of competing interest

The authors declare that they have no known competing financial interests or personal relationships that could have appeared to influence the work reported in this paper.

## Figures and Tables

**Table A1:** Experimental and predicted compressive strengths were calculated using the CNN algorithm.

Water (kg/m³)	Cement (kg/m³)	Fine Aggregate (kg/m³)	Coarse Aggregate (kg/m³)	Admixture (kg/m³)	Experimental Compressive Strength (MPa)	Predicted Compressive Strength (MPa)
197.82	290.92	727.29	972.75	0.00	22.40	22.65
192.94	283.73	709.32	948.72	0.00	21.90	22.23
194.01	285.30	713.26	953.99	0.00	21.75	22.46
190.49	280.14	700.34	936.70	0.00	22.80	23.66
198.88	292.48	731.19	977.97	0.00	21.95	22.44
192.74	283.44	708.60	947.75	0.00	21.85	22.20
193.00	283.82	709.55	949.02	0.00	22.50	22.24
192.08	282.47	706.17	944.50	0.00	21.55	22.17
192.74	283.44	708.60	947.75	0.00	21.95	22.20
192.59	283.21	708.04	947.00	0.00	21.75	22.17
190.90	280.73	701.82	938.69	0.00	22.10	23.00
199.09	292.78	731.96	978.99	0.00	21.95	22.38
199.09	292.78	731.96	978.99	0.00	21.62	22.38
192.74	305.93	725.17	969.91	0.00	25.80	25.91
194.19	308.24	730.65	977.25	0.00	25.60	25.92
194.19	308.24	730.65	977.25	0.00	25.00	25.92
187.23	297.19	704.44	942.19	0.00	25.89	26.97
188.05	298.49	707.53	946.33	0.00	26.62	25.95
187.36	297.40	704.95	942.87	0.00	26.29	26.79
194.19	308.24	730.65	977.25	0.00	26.89	25.92
193.16	306.60	726.76	972.04	0.00	26.31	25.94
194.40	308.57	731.42	978.27	0.00	25.20	25.90
194.19	308.24	730.65	977.25	0.00	26.29	25.92
186.83	296.56	702.95	940.19	0.00	26.72	27.45
189.44	300.69	712.75	953.31	0.00	26.76	25.27
187.36	297.40	704.95	942.87	0.00	26.56	26.79
193.16	306.60	726.76	972.04	0.00	26.20	25.94
182.94	320.94	738.86	988.22	0.00	29.40	29.92
182.74	320.60	738.08	987.18	0.00	29.20	29.87
181.36	318.17	732.48	979.70	0.00	28.60	29.73
176.12	308.99	711.34	951.42	0.00	29.49	29.99
178.41	312.99	720.56	963.75	0.00	30.22	29.97
175.53	307.95	684.33	936.45	0.00	29.89	29.73
180.67	316.96	704.35	963.85	0.00	30.49	29.82
182.02	319.33	709.63	971.07	0.00	29.91	29.35
182.02	319.33	709.63	971.07	0.00	28.80	29.35
182.02	319.33	709.63	971.07	0.00	29.89	29.35
182.02	319.33	709.63	971.07	0.00	30.32	29.35
175.05	307.11	682.47	933.91	0.00	30.36	29.70
177.50	311.40	692.00	946.95	0.00	29.91	29.81
175.53	307.95	684.33	936.45	0.00	28.80	29.73
181.06	317.65	705.88	965.94	0.00	29.89	29.75
182.21	319.66	710.36	972.08	0.00	30.32	29.25
182.02	319.33	709.63	971.07	0.00	30.36	29.35
179.06	316.65	699.88	961.94	0.00	28.60	29.93
176.86	340.12	697.87	954.97	2.57	34.32	35.11
173.96	334.55	686.43	939.33	2.53	34.12	34.61
171.88	330.54	678.21	928.08	2.50	34.12	34.54
176.86	340.12	697.87	954.97	2.57	33.92	35.11
178.17	342.64	703.05	962.06	2.59	35.52	36.03
178.36	343.00	703.77	963.05	2.59	34.17	36.27
178.17	342.64	703.05	962.06	2.59	35.00	36.03
171.42	329.65	676.38	925.58	2.49	35.10	34.53
173.79	334.21	685.75	938.39	2.53	35.48	34.60
178.17	342.64	703.05	962.06	2.59	36.62	36.03
178.17	342.64	703.05	962.06	2.59	36.82	36.03
178.17	342.64	703.05	962.06	2.59	33.82	36.03
173.96	334.55	686.43	939.33	2.53	37.48	34.61
178.17	342.64	703.05	962.06	2.59	35.72	36.03
178.17	342.64	703.05	962.06	2.59	35.52	36.03
178.17	342.64	703.05	962.06	2.59	36.82	36.03
172.65	332.02	681.25	932.23	2.51	34.57	34.56
176.86	340.12	697.87	954.97	2.57	34.57	35.11
178.17	342.64	703.05	962.06	2.59	35.63	36.03
171.88	330.54	678.21	928.08	2.50	35.52	34.54
178.17	342.64	703.05	962.06	2.59	36.62	36.03
171.77	330.33	677.79	927.51	2.50	34.52	34.53
168.56	351.16	684.31	936.43	3.60	36.60	37.70
166.71	347.31	676.81	926.16	3.56	36.40	37.56
172.80	360.00	701.54	960.00	3.69	36.40	39.19
171.53	357.35	696.38	952.94	3.67	36.20	38.35
172.80	360.00	701.54	960.00	3.69	37.80	39.19
172.80	360.00	701.54	960.00	3.69	36.45	39.19
166.61	347.10	676.39	925.59	3.56	37.28	37.55
168.73	351.51	685.00	937.37	3.61	37.38	37.72
166.71	347.31	676.81	926.16	3.56	37.76	37.56
172.80	360.00	701.54	960.00	3.69	37.80	39.19
172.80	360.00	701.54	960.00	3.69	38.90	39.19
172.80	360.00	701.54	960.00	3.69	39.10	39.19
172.80	360.00	701.54	960.00	3.69	36.85	39.19
172.80	360.00	701.54	960.00	3.69	37.91	39.19
167.45	348.86	679.83	930.30	3.58	36.10	37.61
168.56	351.16	684.31	936.43	3.60	36.80	37.70
164.01	381.41	679.18	929.41	3.57	39.76	37.92
162.22	377.26	671.79	919.29	3.54	39.60	37.75
166.88	388.09	691.07	945.67	3.64	40.80	38.67
168.11	390.94	696.15	952.62	3.66	40.20	39.63
168.11	390.94	696.15	952.62	3.66	40.80	39.63
167.23	388.92	692.54	947.69	3.64	39.45	38.87
168.28	391.34	696.86	953.59	3.67	40.28	39.84
168.11	390.94	696.15	952.62	3.66	40.38	39.63
168.11	390.94	696.15	952.62	3.66	40.76	39.63
169.09	393.23	700.23	958.21	3.69	40.80	41.37
168.49	391.83	697.73	954.79	3.67	41.90	40.15
168.44	391.71	697.52	954.49	3.67	42.10	40.07
168.11	390.94	696.15	952.62	3.66	39.85	39.63
167.23	388.92	692.54	947.69	3.64	40.91	38.87
168.11	390.94	696.15	952.62	3.66	42.10	39.63
168.11	390.94	696.15	952.62	3.66	39.80	39.63
168.11	390.94	696.15	952.62	3.66	40.76	39.63
168.11	390.94	696.15	952.62	3.66	40.60	39.63
168.11	390.94	696.15	952.62	3.66	40.80	39.63
168.11	390.94	696.15	952.62	3.66	41.20	39.63
167.50	440.78	682.55	934.02	3.59	48.60	48.43
167.50	440.78	682.55	934.02	3.59	48.60	48.43
167.81	441.59	683.81	935.73	3.60	49.50	48.63
168.61	443.71	687.08	940.21	3.62	50.80	48.17
168.92	444.54	688.36	941.97	3.62	47.60	47.91
168.92	444.54	688.36	941.97	3.62	49.60	47.91
169.09	444.98	689.05	942.91	3.63	45.90	47.77
167.33	440.35	681.88	933.09	3.59	47.10	48.18
168.61	443.71	687.08	940.21	3.62	47.30	48.17
168.61	443.71	687.08	940.21	3.62	47.30	48.17
167.97	442.04	684.49	936.67	3.60	47.40	48.61
168.61	443.71	687.08	940.21	3.62	47.50	48.17
167.33	440.35	681.88	933.09	3.59	47.90	48.18
167.33	440.35	681.88	933.09	3.59	48.50	48.18
167.50	440.78	682.55	934.02	3.59	47.30	48.43
167.97	442.04	684.49	936.67	3.60	47.10	48.61
168.92	444.54	688.36	941.97	3.62	47.30	47.91
224.72	499.38	616.12	843.11	0.00	17.20	15.51
224.72	499.38	616.12	843.11	0.00	15.06	15.51
224.72	499.38	616.12	843.11	0.00	14.07	15.51
224.72	499.38	616.12	843.11	0.00	20.80	15.51
224.72	499.38	616.12	843.11	0.00	20.16	15.51
224.72	499.38	616.12	843.11	0.00	16.88	15.51
224.72	499.38	616.12	843.11	0.00	20.10	15.51
224.72	499.38	616.12	843.11	0.00	20.13	15.51
224.72	499.38	616.12	843.11	0.00	18.80	15.51
224.72	499.38	616.12	843.11	0.00	14.49	15.51
224.72	499.38	616.12	843.11	0.00	13.28	15.51
224.72	499.38	616.12	843.11	0.00	15.02	15.51
224.72	499.38	616.12	843.11	0.00	17.33	15.51
224.72	499.38	616.12	843.11	0.00	16.92	15.51
224.72	499.38	616.12	843.11	0.00	15.89	15.51
224.72	499.38	616.12	843.11	0.00	12.80	15.51
224.72	499.38	616.12	843.11	0.00	12.10	15.51
224.72	499.38	616.12	843.11	0.00	14.20	15.51
224.72	499.38	616.12	843.11	0.00	15.80	15.51
224.72	499.38	616.12	843.11	0.00	16.60	15.51
224.72	499.38	616.12	843.11	0.00	17.10	15.51
224.72	499.38	616.12	843.11	0.00	15.45	15.51
224.72	499.38	616.12	843.11	0.00	14.78	15.51
224.72	499.38	616.12	843.11	0.00	20.40	15.51
224.72	499.38	616.12	843.11	0.00	10.41	15.51
224.72	499.38	616.12	843.11	0.00	10.43	15.51
224.72	499.38	616.12	843.11	0.00	11.46	15.51
224.72	499.38	616.12	843.11	0.00	13.50	15.51
224.72	499.38	616.12	843.11	0.00	9.30	15.51
224.72	499.38	616.12	843.11	0.00	14.60	15.51
224.72	499.38	616.12	843.11	0.00	13.50	15.51
224.72	499.38	616.12	843.11	0.00	14.30	15.51
224.72	499.38	616.12	843.11	0.00	13.60	15.51
224.72	499.38	616.12	843.11	0.00	19.00	15.51
224.72	499.38	616.12	843.11	0.00	20.00	15.51
224.72	499.38	616.12	843.11	0.00	15.30	15.51
224.72	499.38	616.12	843.11	0.00	19.10	15.51
224.72	499.38	616.12	843.11	0.00	20.60	15.51
224.72	499.38	616.12	843.11	0.00	15.19	15.51
224.72	499.38	616.12	843.11	0.00	10.50	15.51
224.72	499.38	616.12	843.11	0.00	11.29	15.51
224.72	499.38	616.12	843.11	0.00	11.28	15.51
224.72	499.38	616.12	843.11	0.00	15.06	15.51
224.72	499.38	616.12	843.11	0.00	15.63	15.51
224.72	499.38	616.12	843.11	0.00	12.66	15.51
